# Going beyond the individual: how state-level characteristics relate to HPV vaccine rates in the United States

**DOI:** 10.1186/s12889-019-6566-y

**Published:** 2019-02-28

**Authors:** Melissa Franco, Stephanie Mazzucca, Margaret Padek, Ross C. Brownson

**Affiliations:** 1 Prevention Research Center in St. Louis, Brown School, Washington University in St. Louis, One Brookings Drive, Campus Box 1196, St. Louis, MO 63130 USA; 2 Department of Surgery (Division of Public Health Sciences), Washington University School of Medicine, Washington University in St. Louis, St. Louis, MO 63103 USA

**Keywords:** Human papillomavirus (HPV), National Immunization Survey-Teen, Cancer prevention, Mis-implementation

## Abstract

**Background:**

The human papillomavirus (HPV) vaccine is an underutilized cancer control practice in the United States. Although individual contextual factors are known to impact HPV vaccine coverage rates, the impact of macro-level elements are still unclear. The aim of this analysis was to use HPV vaccination rates to explore the underuse of an evidence-based cancer control intervention and explore broader-level correlates influencing completion rates.

**Methods:**

A comprehensive database was developed using individual-level date from the National Immunization Survey (NIS)-Teen (2016) and state-level data collected from publically available sources to analyze HPV vaccine completion. Multi-level logistic models were fit to identify significant correlates. Level-1 (individual) and level-2 (state) correlates were fitted to a random intercept model. Deviance and AIC assessed model fit and sampling weights were applied.

**Results:**

The analysis included 20,495 adolescents from 50 U.S. states and the District of Columbia. Teen age, gender, race/ethnicity, and maternal education were significant individual predictors of HPV completion rates. Significant state-level predictors included sex education policy, religiosity, and HPV vaccine mandate. States with the lowest HPV coverage rates were found to be conservative and highly religious. Little variation in vaccine exemptions and enacted sex and abstinence education polices were observed between states with high and low HPV vaccine coverage suggesting various contextual and situational factors impact HPV vaccine completion rates.

**Conclusions:**

Given that gender, religiosity, political ideology, and education policies are predictors of HPV vaccine completion, the interaction and underlying mechanism of these factors can be used to address the underutilization of the HPV vaccine.

## Background

In the United States, human papillomavirus (HPV) is the most common sexually transmitted infection causing genital warts and HPV-related cancers [1]. HPV transmission can occur through vaginal, anal, or oral sex with an HPV-infected individual [1]. As symptoms can develop years after infected with HPV, onset of the virus can be difficult to diagnosis [1]. Currently, about 79 million people are infected with HPV and an additional 14 million cases are projected annually in the United States [1]. Each year, about 30,000 Americans develop cancer as a result of an HPV infection with incidence and prevalence of HPV-related cancers disproportionately higher in Hispanic and African American woman compared to their counterparts [2–4].

Efficacy studies have indicated HPV vaccines are a highly effective prevention tool prior to HPV exposure [5, 6]. As a result, the Advisory Committee on Immunization Practices (ACIP) recommends routine vaccinations for all children and adolescents beginning as early as age nine [7]. Vaccines are known to be one of the few highly effective public health strategies that address many preventable diseases, such as HPV-related cancers and infections [8]. Gradual progress to improve access to HPV vaccines and initiatives to increase vaccine uptake have been seen in recent years. Efforts include: 1) communication campaigns and strategies, 2) mandating private and public insurance plans to provide coverage for preventative services, including the HPV vaccine and testing, under the Affordable Care Act and Medicaid Expansion, 3) distribution of HPV vaccines to uninsured and low-income individuals by the Vaccine for Children program, and 4) improving health through benchmarks such as Healthy People 2020, which sets to achieve an 80% HPV vaccine coverage rate among U.S. adolescents [9–11]. Yet, HPV vaccination coverage is drastically lower than other childhood vaccines with a 2017 national average of 43%, including males and females, compared to a coverage of 91.1% for measles, mumps, and rubella (MMR) vaccine [12, 13].

Attitudes and beliefs that HPV is a low health priority and vaccination is an elective health measure have resulted in low adherence to HPV vaccine guidelines [14]. Considerable attention on individual, provider, and family contextual factors explaining low HPV adherence (e.g., vaccine safety and efficacy knowledge), acceptability (e.g., parental intent to vaccinate), and barriers (e.g., providers informed of HPV vaccine schedule/updates) has been observed in the literature [14–18]. Reliance on high-risk strategies for the HPV vaccine (e.g., identify genetic dispositions and disease risk/development) to emphasize prevention and screening, specifically among those at high-risk of an HPV infection, has been abandoned as these efforts are unsuccessful and high cost with limited potential [19, 20]. A shift to a population-based strategy (e.g., adopted normality of seat belt usage) will focus HPV vaccine coverage efforts on broader-level factors to reduce the risk of HPV infections by altering behavior norms and have a wider reach [19].

In order to explore opportunities to enhance HPV vaccine uptake, the aim of this paper is to study the combination of individual (e.g., gender, race) and macro-level factors (e.g., political environment, state religiously, sex/abstinence education polices) potential influences on HPV vaccine completion rates as limited evidence is available. Further understanding these relationships, will not only broaden the evidence in the literature to consider influences beyond the individual, but provide guidance and support to leaderships and facilitators whom have decision-making power in HPV vaccine-related activities. These findings can potentially translate into successful implementation of HPV vaccine interventions and identify potential influences hindering their utilization. We hypothesize that broader-level factors, in addition to individual-level influences, highlights potential modifiable and non-modifiable HPV vaccine barriers influencing suboptimal HPV vaccine adherence nationally. More specifically, we seek to (1) explore possible factors of high and low HPV vaccine coverage rates, and (2) illustrate the current HPV vaccine coverage rates across the United States.

## Methods

For our analyses, a comprehensive database was created using individual and state-level variables from publicly available sources.

### Data sources

#### Individual-level data

The National Immunization Survey (NIS)-Teen (2016) is a cross-sectional survey which estimates vaccine coverage rates among 13–17 year old teens in the United States using random digit dialing conducted by the National Center for Immunization and Respiratory Diseases and the National Center for Health Statistics within the Centers for Disease Control and Prevention (CDC) [21, 22]. Telephone interviews were conducted with parents regarding their teen’s vaccination history and teen and family sociodemographic factors (e.g., teen age and maternal education). With parental consent, the teen’s medical provider(s) were contacted to request medical records to verify vaccine history [21, 22]. We excluded teens without medical provider-confirmed vaccination history [22]. The NIS-Teen was approved by the Ethics Review Board of the National Center for Health Statistics, Centers for Disease Control and Prevention.

#### State-level data

Funding appropriated for HPV vaccination promotion efforts in 2017 and state and local public health governance (2015) was taken from the Centers for Disease Control and Prevention (CDC) websites [23, 24]. The 2010 state population was obtained from the United States Census Bureau webpage [25]. State-wide policies related to sexual education and vaccination requirements were taken from the Guttmacher Institute (2017) and the Immunization Action Coalition (2017), respectively [26, 27]. Religious and political opinions by state were available from the Pew Research Center (2014) [28, 29]. Individual and state-level data were obtained at the most recent report for each source at the time of analysis.

### Outcome specification

Vaccine coverage, or completion data, was based on medical records from providers and vaccines administered after the NIS-Teen interview were excluded [22]. HPV vaccine completion was defined as having ≥3 HPV shots or ≥ 2 HPV shots with the first shot received before age 15 and the interval between the 1st and 2nd shots at least 5 months and 4 days apart. Teens were considered up to date on their Tdap (Tetanus, Diphtheria, and Pertussis) vaccination completion if they had received ≥1 shot since age 10 and for MMR vaccine if they had received ≥2 shots at the time of the survey.

### Exposure specification

#### Teen and family correlates

Covariates at the teen and family-level were chosen from the NIS-Teen dataset based on previous literature and included teen age, gender, race/ethnicity; maternal education; family poverty status; and whether a provider had recommended an HPV vaccine series for the child. Indicator variables were used to examine race and ethnicity of the teen as Non-Hispanic White, Non-Hispanic Black, Hispanic, and Non-Hispanic other or mixed race. Poverty status of the family was categorized as above or below the federal poverty line, and maternal education was dichotomized as up to a college graduate and college graduate or higher.

#### State correlates

State public health departments were categorized according to their jurisdiction type as decentral**,** central, mixed, or shared [24]. Funding was determined in millions of U.S. dollars from several CDC funding lines that could be used towards HPV vaccine promotion efforts: immunization & respiratory disease; vaccines for children; HIV/AIDS and viral hepatitis; sexually transmitted infection and tuberculosis prevention; chronic disease prevention and health promotion, and cancer prevention control. CDC funding per capita was calculated as the total amount of CDC funding in the previously identified categories divided by the 2010 population.

The presence of an HPV vaccine and sex education mandate in state legislation, along with the type of abstinence-only policy (e.g., whether abstinence was covered or stressed) were also examined as potential state correlates. States that have “stressed abstinence” policies strongly emphasizes abstinence as a standard teaching [30]. Alternatively, “covered abstinence” education addresses abstinence and also includes information about contraception [30]. Political ideology was categorized as percent of the state identifying as conservative, moderate, liberal, or those who did not know their political ideology. Additionally, the religious tendency of a state was expressed as the percent of the state identifying as highly religious [29].

### Statistical analysis

Multi-level models were fit to identify important correlates of HPV vaccine completion and quantify relationships with odds ratios and 95% confidence intervals (CI). First an unconditional logistic model was fit without any independent variables to calculate the intra-class correlation for states. Random intercept models were then fit with individual (level-1) and subsequently state (level-2) correlates. Model fit was assessed using deviance and Akaike information criterion (AIC). Sampling weights calculated for the provider-confirmed data were taken from the NIS-Teen data, which account for selection and non-response probability and were rescaled to account for the multi-level nature of the data according to best practice recommendations [31]. Models were fit with and without rescaled sampling weights, and models presented are those fit with sampling weights that were rescaled to add up to the cluster size. There were no missing data once the outcome variable, provider confirmed receipt of HPV vaccine, was selected.

Additionally, an analysis of education policy and broader-level factors on HPV vaccine completion rates was done. Sex and abstinence education policies were coded into four groups based on comprehensiveness of policy: (1) having both a sex and abstinence education policy (SA-YY), (2) having a sex education only policy and not an abstinence education policy (SA-YN), (3) not having a sex education policy and having an abstinence only education (SA-NY), and (4) not having either a sex and abstinence education policy (SS-NN) (Table 3). Vaccine exemptions were also coded into three subgroups: (1) medical vaccine exemption (M), (2) medical and religious vaccine exemption (MR), and (3) medical, religious, and personal vaccine exemption (MRP). HPV vaccine completion rates, religiosity, political ideology, and vaccine exceptions by high and low state vaccine implementers were also analyzed. A national map to visualize state-specific HPV vaccine completion was also done. All statistical analyses were performed in SAS 9.4 (Cary, NC), MPlus Version 8, and ArcMap 10.5.1 [32].

## Results

The 2016 NIS-Teen survey included 20,495 teens with provider-confirmed vaccination history and were included in our analyses, which was 49% of the interviewed sample. The average adolescent age was 15 years old with the majority of teens being Non-Hispanic White and female (Table 1). State-level data included the 50 U.S. states and the District of Columbia. Table 2 illustrates that currently only the District of Columbia and two states had enacted an HPV vaccine mandate (Rhode Island and Virginia) with the majority in support of abstinence education (Table 3). Most states identified as highly religious and had a conservative political ideology. Few had a centralized public health governance with the majority of public health departments being decentralized in the United States. Further, about $43 million was potentially available for HPV vaccine promotion efforts with a CDC funding per capita of approximately $17 in 2016.

**Table 1:** Teen- and Family-level Characteristics

	Mean	SD
Teen age (years)	15.0	1.4
	N	%
HPV vaccine completion	9204	43.4
Teen race/ethnicity		
Non-Hispanic White	12,883	63%
Non-Hispanic Black	1990	10%
Hispanic	3223	16%
Non-Hispanic other or mixed race	2379	12%
Teen gender – Female	9661	47%
Family below poverty line	3461	17%
Mother is a college graduate	9654	47%

**Table 2:** State-level Characteristics

	N	%
HPV mandate present	3	6%
Sex education policy present	25	56%
Abstinence only education policy present	37	88%
Stressed	26	70%
Covered	11	30%
Public Health Department Type		
Decentral	28	55%
Central	13	25%
Mixed	5	10%
Shared	5	10%
	Mean	SD
Percent of state identifying as highly religious	54.67	10.64
Political Ideology		
Conservative	36.92	0.07
Moderate	33.39	0.03
Liberal	23.35	0.06
Don’t Know	6.27	0.02
Center for Disease Control and Prevention Funding		
Funding per capita (USD)	$17.34	$21.72
Total CDC Funding (millions USD)	67.56	60.61
Immunization & Respiratory Disease	7.02	6.89
Vaccine for Children	1.87	1.78
HIV/AIDS, Viral Hepatitis, STI and TB Prevention	14.01	20.97
Chronic Disease Prevention and Health Promotion	14.71	10.27
Cancer Prevention Control	5.40	3.21

**Table 3:** State HPV Coverage Rates and Sex and Abstinence Education Polices

Education Policy Sets^a^	N (%)	% HPV Vaccine Completion (SD)
Y-Sex, Y-Abstinence	22 (43)	45.0 (10.26)
Y-Sex, N-Abstinence	3 (6)	49.1 (11.49)
N-Sex, Y- Abstinence	15 (29)	40.9 (5.58)
N-Sex, N-Abstinence	11 (22)	44.6 (9.37)

^a^*Y* Yes Policy Present, *N* No Policy Present

**Fig. 1:**
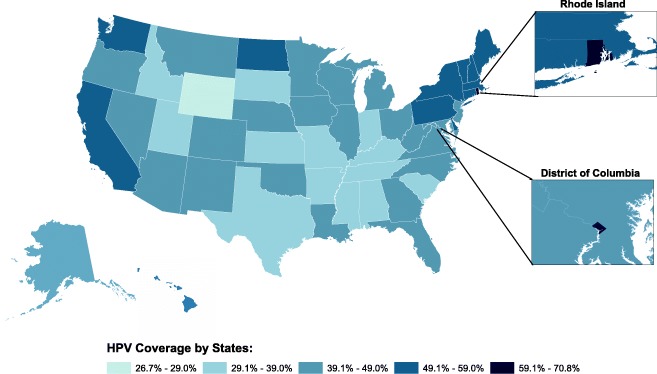
HPV vaccine completion by state, 2016

Rhode Island (70.8%) and the District of Columbia (62.0%) had the highest HPV vaccine coverage rates in the United States. Those with the lowest HPV vaccine coverage rates in 2016 were Wyoming, Mississippi, South Carolina, Utah, and Texas. These states were found to be more conservative and highly religious than states with the highest coverage rates. Little variation among these states regarding education polices and vaccine exemptions was observed. (Table 4 and Fig. 1).

**Table 4:** Top 5 High and Low HPV Coverage States^a^

State or District	Religiosity (%)	Political Ideology			Education Policy Sets^b^	Vaccine Exemption^c^	HPV vaccine completion (%)
		Conservative (%)	Moderate (%)	Liberal (%)			
High State HPV Coverage Rates							
RI	49	26	35	30	SA-YY	MR	70.8
DC	53	15	39	36	SA-YN	MR	62.0
DE	52	26	40	25	SA-YY	MR	56.9
MA	33	23	35	36	SA-NN	MR	56.6
ME	34	34	33	30	SA-YY	MRP	56.0
Low State HPV Coverage Rates							
TX	64	39	32	21	SA-NY	MRP	32.9
UT	64	45	31	20	SA-YY	MRP	30.5
MS	77	45	30	19	SA-YY	M	29.1
SC	70	43	35	15	SA-YY	MR	29.1
WY	54	41	33	17	SA-NN	MR	26.7

^a^Overview of the states with the highest and lowest HPV completion rates in the United States and current comparison of political ideology, religiosity, education policy, and vaccine exemptions per state

^b^*SA-YY* sex-YES & abstinence-YES, *SA-YN* Sex-YES & abstinence-NO, *SA-NY* Sex-NO & abstinence-YES, *SA-NN* Sex-NO & abstinence-NO

^c^*M* Medical vaccine exemption, *MR* Medical and religious vaccine exemption, *MRP* Medical, religious, and personal vaccine exemptio

As shown in Table 5, individual and state effects of HPV vaccine completion were teen gender-female (OR, 1.65; 95% Cl,1.56–1.75), Hispanic teen race/ethnicity (OR, 1.52; 95% Cl,1.36–1.69), Non-Hispanic Black teen race/ethnicity (OR, 1.34; 95% Cl, 1.18–1.51), HPV vaccination recommended by healthcare provide (OR, 3.57; 95% 3.24–3.92), and HPV vaccine mandate (OR, 1.65; 95% Cl 1.16–2.35). Abstinence education, CDC funding (per capita), and public health governance were found to be non-significant model factors. Among teens, Hispanics/Blacks, females, and those living below the poverty line were more likely to complete the HPV vaccine series compared to their counterparts.

**Table 5:** Associations between individual and state-level factors and vaccine completion

	Full model				Reduced model^a^			
	OR	95% CI		*p*-value	OR	95% CI		*p*-value
Teen age	1.17	1.13	1.21	< 0.0001	1.17	1.13	1.21	< 0.0001
Teen race/ethnicity								
Non-Hispanic White (ref)	1				1			
Non-Hispanic Black	1.41	1.24	1.62	< 0.0001	1.42	1.24	1.62	< 0.0001
Hispanic	1.61	1.45	1.79	< 0.0001	1.61	1.45	1.79	< 0.0001
Non-Hispanic other or mixed race	1.27	1.15	1.40	< 0.0001	1.27	1.15	1.40	< 0.0001
Teen gender – Female	1.50	1.41	1.60	< 0.0001	1.50	1.41	1.60	< 0.0001
Family below poverty line	1.61	1.47	1.77	< 0.0001	1.61	1.47	1.77	< 0.0001
Mother is a college graduate	1.13	1.04	1.23	0.018	1.13	1.04	1.23	0.017
Provider recommended HPV vaccine	3.56	3.24	3.92	< 0.0001	3.57	3.24	3.92	< 0.0001
HPV mandate present	1.55	1.02	2.36	0.082	1.71	1.19	2.45	0.015
Percent of state identifying as highly religious	0.13	0.06	0.26	< 0.0001	0.13	0.08	0.20	< 0.0001
Sex education policy present	1.09	0.96	1.23	0.269				
Percent of state identifying as conservative	0.67	0.23	1.99	0.545				
Abstinence only education policy absent (ref)	1							
Abstinence stressed	1.05	0.91	1.21	0.576				
Abstinence covered	1.09	0.91	1.23	0.437				
CDC funding per capita (USD)	1.00	0.99	1.02	0.726				
Public Health Department Type								
Decentral (ref)	1							
Central	1.06	0.91	1.23	0.509				
Mixed	1.07	0.82	1.39	0.693				
Shared	1.01	0.84	1.21	0.939				

^a^Logistic regression models were fit to quantify the relationships between individual- and state-level factors and HPV vaccine completion. The full model represents all correlates that were examined, and the reduced model includes only those correlates that were statistically significantly associated (*p* < 0.05) with HPV vaccine completion

*OR* Odds ratio, *CI* Confidence interval

Additionally, states with sex education polices and an HPV vaccine mandate were found to have higher completion rates. A larger percentage of highly religious adults within a state had significantly lower rates of HPV vaccine completion (with by far, the largest effect estimate). Gender, political ideology, religiosity, and sex education policy were not found to have similar impacts on Tdap and MMR vaccination rates as the HPV vaccine. See Table 5 for other significant predictors of HPV vaccine completion.

## Discussion

This multi-level analysis using state and national data found the significant predictors of HPV vaccine completion were the presence of an HPV vaccine mandate, religiosity, being female, and Hispanic or Black race/ethnicity. Although we expect race, gender, living below the poverty line, and maternal college education to be indicative of higher HPV vaccine completion rates, the effects of additional macro-level factors are important to consider in improving suboptimal HPV vaccine adherence. This suggests that a state’s sex and abstinence education policy, religiosity, and political ideology may be important correlates of HPV vaccine completion.

Our analysis suggests an 11-fold decrease in HPV vaccine completion rates among states with a higher percentage of religious adults. Religion has been found to influence immunization decisions as several states have religious exemptions for school mandated vaccines. If parents are religiously opposed to immunizing their children against infectious disease such as tetanus or measles, the likelihood of vaccinating these teens for a sexually transmitted disease like HPV poses a challenge to vaccine completion rates. Although there is limited evidence, the role of religion has been found to guide decisions around certain health behaviors therefore influencing HPV vaccine acceptance and uptake [33, 34]. As most literature focuses on the intent to vaccinate teens against HPV based on religious beliefs, few give insights on religious impact on HPV vaccine completion.

A study in Utah found that non-religious females were more likely to complete the HPV vaccine series (received 3 doses) than religious females [34]. A similar trend is found in our study sample as states that had a higher percentage of religious adults showed lower HPV vaccine completion rates. By considering the religious climate, states with a denser religious population (e.g., Utah) can benefit from faith-based HPV vaccine interventions delivered by religious leaders to increase coverage rates. For example, Body & Soul, a faith-based intervention, sought to increase vegetable and fruit consumptions among African Americans by partnering with churches to encourage diet change [35]. By taking this approach, challenges faced by current traditional interventions targeted towards the general public can be overcome to improve HPV knowledge, awareness, and acceptance as well as HPV vaccine coverage among religious communities.

Little is known about how strongly and consistently state-level polices impact HPV health outcomes, but these results indicate certain policies may impact HPV vaccine coverage rates. There is significant political opposition and criticism to enact legislation mandating HPV vaccination for adolescents [36, 37]. As a result, only two states and the District of Columbia have successfully passed legislation requiring teens to be vaccinated [37]. Dempsey et al. suggests the use of stringent school mandates can improve HPV vaccine completion [38]. They predict a 90% completion with the presence of a school mandate can be achieved in 50 years opposed to a 79% completion in 43 years without a school mandate in place [38]. In the United States, requiring other childhood vaccines (MMR, Tdap) for school enrollment has been successful in maintaining coverage rates, but we are missing the opportunity to widely accept and use school-mandates for the HPV vaccine. Ineffective policy implementation is limiting the potential of a known evidence-based intervention to prevent HPV-related infections and cancers. Our results suggest that suboptimal utilization of the HPV vaccine may be increased through HPV school-mandates by requiring teens to be vaccinated for school enrollment.

Despite strong support for abstinence-only education policies among some stakeholders, [39] it has been found that they do not delay sexual initiation, prevent teen pregnancies, number of sexual encounters, or reduce STI rates [40, 41]. Although enactment of sex education-only policies showed significance on HPV vaccine completion rates in our analyses, on average, states with both sex and abstinence education policies had a slightly higher HPV coverage rate compared to states that had not enacted any education policies. This finding suggests that other contextual factors are driving HPV vaccine completion in states that do not have either policy in place.

Furthermore, as shown in other studies, [42–45] our results indicate females are more likely than males to complete the HPV vaccine series. There are several factors to consider that are resulting in higher HPV vaccine completion rates among females. Initially, the identification of HPV as a gender-specific disease has led to a feminization of the HPV vaccine, contributing parental opposition to vaccinate their male children [42, 46]. Although both genders are at risk for HPV, it is estimated that males are more likely than females to contract an HPV infection in their lifetime [4, 42]. Despite this, the burden of HPV screening, vaccination, and treatment falls heavily on woman [42, 46]. Several efforts are shifting the mentality of HPV as a female-specific disease, including media campaigns that incorporate messages around male vaccination, the addition of males to the ACIP HPV vaccine recommendations in 2010, and a growing body of evidence of the burden of disease in both males and females [37]. Further, our results strongly indicate that healthcare providers play a major role in HPV vaccine completion rates. Gender bias among healthcare providers’ recommendations could also be influencing HPV vaccination rates among males [42, 45]. As HPV vaccine recommendations by providers has been shown to influence parental acceptance to vaccinate their teen, there is a discrepancy in receiving HPV vaccine recommendations by gender [45, 47]. Parents report healthcare providers are less likely to recommend the HPV vaccine if their child is male compared to a female child indicating providers need to be aware of the potential effects of these biases [45, 48]. This further stress the importance of provider recommendation for HPV vaccine completion, regardless of gender, to achieve desired coverage rates. Altogether, these factors may explain situational factors influencing parental decisions to vaccinate male teens.

There are many factors that affect HPV vaccine completion rates as is not reliant on a single policy or individual-level characteristics. Gender, religiosity, political ideology, and education polices are independent predictors of HPV vaccine completion rates as they were not found to be present in Tdap and MMR vaccine completion models. Thus, further research should consider the complexity of the political and religious environment as well as other state-level policies must be considered when trying to the address the underutilization of this cancer prevention vaccine. Roberts et al. identified a combination of state-level policies associated with higher vaccine uptake, including Medicaid expansion, HPV vaccine school mandates, HPV vaccination through pharmacies, and sex education policies [36]. This suggests that specific policies have significant influences on HPV vaccine adherence and their interactions within states is needed to understand the contextual and situational factors that contribute or prevent a teen from completing the HPV vaccine series.

This analysis can also serve as an example showing how mis-implementation of an evidence-based cancer intervention is occurring. According to Padek et al., mis-implementation is the continuation of ineffective programs and policies or the discontinuation of effective ones [49]. The implementation of evidence-based interventions such as the HPV vaccine has the potential to prevent HPV-related cancers and infections. However, the underuse of this population-based intervention has not achieved their potential effect on HPV-related cancer outcomes. This could perhaps be explained by the discontinuation of this evidence-based intervention at the individual and state-level. There could be a variety of factors at play on why these programs or policies are discontinued or not implemented at all and a better understanding of the demographics which underutilize this vaccine could help facilitate more successful implementation of programs or policies.

Next steps should explore current HPV vaccine-related policies and initiatives in states not having neither education policy to gain insight into other activities contributing to HPV vaccine completion rates. As states that enacted sex education policies were found to have the highest coverage rates among any policy combination, more study of the effects of comprehensive sex education to increase HPV vaccine coverage rates is supported.

Although the governance of state health departments were not significant predictors of HPV vaccine completion rates, research to understand the role of state health departments on current efforts, barriers, and allocation of resources regarding cancer control programs is essential to identify the mis-implementation at a state-level. Considering and understanding the role of factors leading to mis-implementation of cancer control programs and policies regarding HPV vaccine can prevent missed opportunities to appropriately implement these efforts.

Lastly, learning and further exploring elements that contribute to low and high vaccine coverage rates among states can inform the development of comprehensive HPV vaccine interventions and policies to increase the utilization of the HPV vaccine in the United States.

### Limitations

We acknowledge that there are several limitations to this analysis. The cross-sectional nature of our study limits our ability to draw any firm conclusions on the macro-level contributing factors to low-HPV vaccine uptake. Evidence shows that provider-level recommendations has a significant influence on patient uptake of the vaccine [50–52].However, given other macro-level factors, provider recommendations may vary greatly by geographic region. This would be pertinent to explore further in future analyses. Further, while the use of a nationally representative sample is a major strength of this analysis, the pooling of various sources limits the generalizability of results and cannot be used to predict future HPV vaccine completion rates.

## Conclusion

Despite the study’s limitations, this analysis highlights the potential impacts of broader contextual factors effects on HPV vaccine coverage rates in the United States. found that in addition to individual-level correlates (e.g., teen age, race, socioeconomic status), policy, political ideology, and religiosity state-level factors are significant correlates of HPV vaccine completion rates. However, there is limited evidence on how these factors interact with each other within a state to promote HPV vaccine efforts. Further studies need to continue to identify state and local-level factors that contribute to HPV vaccine uptake and adherence as well as understand the underlying mechanisms of these factors within states of high and low HPV vaccine coverage rates. An in-depth case analysis of these states may provide insight on effective strategies, initiatives, and policies along with overcome challenges and barriers to serve as a guidance for other states and better inform HPV vaccine interventions.

## Abbreviations

ACIP: Advisory committee on immunization practice
CDC: Centers for disease control and prevention
HPV: Human papillomavirus
MMR: Measles, mumps, and rubella
NIS: National immunization survey
Tdap: Tetanus, diphtheria, and pertussis

## References

[1] Centers for Disease Control and Prevention. Genital HPV Infection-Fact Sheet. https://www.cdc.gov/std/hpv/stdfact-hpv.htm. Updated November 16, 2017.

[2] Centers for Disease Control and Prevention. Human Papillomavirus (HPV) Vaccination & Cencer Prevention. https://www.cdc.gov/vaccines/vpd/hpv/index.html. Updated November 22, 2016.

[3] Centers for Disease Control and Prevention. HPV-Associated Cancer Rates by Race and Ethnicity. https://www.cdc.gov/cancer/hpv/statistics/race.htm. Updated August 15, 2018.

[4] McQuillan G, Kruszon-Moran D, Markowitz L, Unger E, Paulose-Ram R (2017). Prevalence of HPV in adults aged 18–69: United States, 2011–2014. NCHS Data Brief vol. no 280.

[5] Markowitz L, Dunne E, Saraiya M, Chesson H, Curtis C, Gee J, Bocchini J Jr, Unger E. Human Papillomavirus Vaccination Recommedantions for the Advisiry Committee on Immunization Practices (ACIP). Morb Mortal Wkly Rep. 2014;63(RR05):1-30 25167164

[6] Centers for Disease Control and Prevention. Human Papillomavirus (HPV) Vaccine. https://www.cancer.gov/about-cancer/causes-prevention/risk/infectious-agents/hpv-vaccine-fact-sheet#q8. Updated May 16, 2018.

[7] Meites E, Kempe A, Markowitz LE. Use of a 2-Dose Schedule for Human Papillomavirus Vaccination — Updated Recommendations of the Advisory Committee on Immunization Practices. MMWR Morb Mortal Wkly Rep. 2016;65:1405–8. 10.15585/mmwr.mm6549a5. 10.15585/mmwr.mm6549a5 27977643

[8] Centers for Disease Control and Prevention. Ten great public health achievements—United States, 1900–1999. MMWR Morb Mortal Wkly Rep. 1999;48:241–243. 10220250

[9] Kaiser Family Foundation. The HPV Vaccine: Access and Use in the U.S. https://www.kff.org/womens-health-policy/fact-sheet/the-hpv-vaccine-access-and-use-in/. October 9, 2018.

[10] Healthy People 2020. Immunization and Infectious Diseases. https://www.healthypeople.gov/2020/topics-objectives/topic/immunization-and-infectious-diseases/objectives. Updated February 21, 2019.

[11] HPV Vaccination for Cancer PRevention (2018). Progress, Opportunities, and a Renewed Call to Action. A Report to the President of the United States from the Chair of the President's Cancer Panel.

[12] Walker TY, Elam-Evans L, Yankey D, Markowitz L, Williams C, Mbaeyi S (2018). National, regional, State, and selected local area vaccination coverage among adolescents aged 13–17 years — United States, 2017. MMWR Morb Mortal Wkly Rep.

[13] Hill H, Elam-Evans L, Yankey D, Singleton J, Yoonjae K (2017). Vaccination coverage among children aged 19–35 months — United States, 2016. MMWR Morb Mortal Wkly Rep.

[14] Chuang E, Cabrera C, Mak S, Glenn B, Hochman M, Bastani R (2017). Primary care team- and clinic level factors affecting HPV vaccine uptake. Vaccine.

[15] Allen JD, Othus MK, Shelton RC, Li Y, Norman N, Tom L (2010). Parental decision making about the HPV vaccine. Cancer Epidemiol Biomarkers Prev.

[16] Hershey JH, Velez LF (2009). Public health issues related to HPV vaccination. J Public Health Manag Pract.

[17] Rand CM, Schaffer SJ, Humiston SG, Albertin CS, Shone LP, Heintz EV (2011). Patient—provider communication and human papillomavirus vaccine acceptance. Clin Pediatr.

[18] Reiter PL, Brewer NT, Gottlieb SL, McRee A-L, Smith JS (2009). Parents’ health beliefs and HPV vaccination of their adolescent daughters. Soc Sci Med.

[19] Rose G (2001). Sick individuals and sick populations. Int J Epidemiol.

[20] Brewer NT, Calo WA (2015). HPV transmission in adolescent men who have sex with men. Lancet Infect Dis.

[21] Centers for Disease Control and Prevention. About the National Immunization Surveys. https://www.cdc.gov/vaccines/imz-managers/nis/about.html. Updated December 27, 2018.

[22] Centers for Disease Control and Prevention. National Immunization Survey-Teen A User's Guide for the 2016 Public-use data file. https://www.cdc.gov/vaccines/imz-managers/nis/downloads/NIS-TEEN-PUF16-DUG.pdf. National Center for Immunization and Respiratory Diseases. October 2017. p. 1–219.

[23] Centers for Disease Control and Prevention. Grant Funding Profiles- Summary View. 2017. https://wwwn.cdc.gov/FundingProfilesApp.

[24] Centers for Disease Control and Prevention. Health Department Governance. https://www.cdc.gov/stltpublichealth/sitesgovernance. Updated October 23, 2018.

[25] United States Census Bureau. Quick Facts United States. 2017. https://www.census.gov/quickfacts/fact/table/US/PST045217.

[26] Guttmacher Institute. Sex and HIV Education. 2018. https://www.guttmacher.org/state-policy/explore/sex-and-hiv-education.

[27] Immunization Action Coalition. State Information HPV Mandates for Children Secondary Schools. http://www.immunize.org/laws/hpv.asp. Updated November 11, 2018.

[28] Pew Research Center. Political Ideology by State. 2014. http://www.pewforum.org/religious-landscape-study/compare/political-ideology/by/state/.

[29] Pew Research Center. How religious is your state? http://www.pewresearch.org/fact-tank/2016/02/29/how-religious-is-your-state/?state=alabama. Updated February 29, 2016.

[30] Editorial Projects in Education. Abstinence Education in State Laws. https://www.edweek.org/rc/articles/2007/06/08/sow0608.h26.html. June 8, 2007.

[31] Carle AC (2009). Fitting multilevel models in complex survey data with design weights: recommendations. BMC Med Res Methodol.

[32] Muthén L, Muthén B (1998). Mplus User’s guide, eighth edition edn.

[33] Shelton RC, Snavely AC, De Jesus M, Othus MD, Allen JD (2013). HPV vaccine decision-making and acceptance: does religion play a role?. J Relig Health.

[34] Bodson J, Wilson A, Warner EL, Kepka D (2017). Religion and HPV vaccine-related awareness, knowledge, and receipt among insured women aged 18-26 in Utah. PLoS One.

[35] De Marco M, Weiner B, Meade SA, et al. Assessing the readiness of black churches to engage in health disparities research. J Natl Med Assoc. 2011;103(9-10):960-7. 10.1016/s0027-9684(15)30453-3 PMC3296968 22364066

[36] Roberts MC, Murphy T, Moss JL, Wheldon CW, Psek W (2018). A qualitative comparative analysis of combined State health policies related to human papillomavirus vaccine uptake in the United States. Am J Public Health.

[37] Keim-Malpass J, Mitchell EM, DeGuzman PB, Stoler MH, Kennedy C (2017). Legislative activity related to the human papillomavirus (HPV) vaccine in the United States (2006–2015): a need for evidence-based policy. Risk Manage Healthcare Policy.

[38] Dempsey AF, Mendez D (2010). Examining future adolescent human papillomavirus vaccine uptake, with and without a school mandate. J Adolesc Health.

[39] Casper MJ, Carpenter LM (2008). Sex, drugs, and politics: the HPV vaccine for cervical cancer. Sociol Health Illn.

[40] Santelli J, Ott MA, Lyon M, Rogers J, Summers D, Schleifer R. Abstinence and abstinence-only education: A review of U.S. policies and programs. J Adolesc Health. 2006;38(1):72–81. 10.1016/j.jadohealth.2005.10.006. 10.1016/j.jadohealth.2005.10.006 16387256

[41] Charo RA (2007). Politics, parents, and prophylaxis — mandating HPV vaccination in the United States. N Engl J Med.

[42] Daley EM, Vamos CA, Thompson EL, Zimet GD, Rosberger Z, Merrell L (2017). The feminization of HPV: how science, politics, economics and gender norms shaped U.S. HPV vaccine implementation. Papillomavirus Res.

[43] Choi Y, Eworuke E, Segal R (2016). What explains the different rates of human papillomavirus vaccination among adolescent males and females in the United States?. Papillomavirus Res.

[44] Voss DS, Wofford LG (2016). Human papillomavirus vaccine uptake in adolescent boys: an evidence review. Worldviews Evid-Based Nurs.

[45] Johnson KL, Lin M-Y, Cabral H, Kazis LE, Katz IT (2017). Variation in human papillomavirus vaccine uptake and acceptability between female and male adolescents and their caregivers. J Community Health.

[46] Hull SC, Caplan AL (2009). The case for vaccinating boys against human papillomavirus. Public Health Genomics.

[47] Alice Yuen L, Miu Ling K, Yuen-Ting W, Alice Kar Yan W (2017). The uptake of human papillomavirus vaccination and its associated factors among adolescents: a systematic review. J Prim Care Community Health.

[48] Malo TL, Giuliano AR, Kahn JA, Zimet GD, Lee JH, Zhao X (2014). Physicians’ human papillomavirus vaccine recommendations in the context of permissive guidelines for male patients: a national study. Cancer Epidemiol Biomarkers Prev.

[49] Padek M, Allen P, Erwin PC, Franco M, Hammond RA, Heuberger B (2018). Toward optimal implementation of cancer prevention and control programs in public health: a study protocol on mis-implementation. Implement Sci.

[50] Dorell C, Yankey D, Kennedy A, Stokley S (2012). Factors that influence parental vaccination decisions for adolescents, 13 to 17 years old: National Immunization Survey–Teen, 2010. Clin Pediatr.

[51] Kessels SJM, Marshall HS, Watson M, Braunack-Mayer AJ, Reuzel R, Tooher RL (2012). Factors associated with HPV vaccine uptake in teenage girls: a systematic review. Vaccine.

[52] Reiter PL, McRee A-L, Pepper JK, Gilkey MB, Galbraith KV, Brewer NT (2013). Longitudinal predictors of human papillomavirus vaccination among a National Sample of adolescent males. Am J Public Health.

